# (−)-Epicatechin Ameliorates Monosodium Urate-Induced Acute Gouty Arthritis Through Inhibiting NLRP3 Inflammasome and the NF-κB Signaling Pathway

**DOI:** 10.3389/fphar.2022.799552

**Published:** 2022-04-06

**Authors:** Chenxi Wu, Fenfen Li, Xiaoxi Zhang, Wenjing Xu, Yan Wang, Yanjing Yao, Ziwei Han, Daozong Xia

**Affiliations:** ^1^ School of Pharmaceutical Sciences, Zhejiang Chinese Medical University, Hangzhou, China; ^2^ Academy of Chinese Medical Sciences, Zhejiang Chinese Medical University, Hangzhou, China

**Keywords:** anti-inflammatory, (−)-epicatechin, gouty arthritis, NF-κB signaling pathway, NLRP3 inflammasome

## Abstract

**Background:** Gouty arthritis is a common and complex inflammatory disease that will reduce the life quality of human beings (−)-Epicatechin (EC) is famous for antioxidant and anti-inflammatory activities. Thus, the aim of this study was to investigate the therapeutic effect of EC on gouty arthritis and its mechanisms.

**Methods and results:** EC was added into a monosodium urate (MSU)-stimulated THP-1 cell that was induced by phorbol 12-myristate 13-acetate and lipopolysaccharide (LPS) in advance to establish a gout model *in vitro*. The efficiency of EC on acute gouty arthritis mice induced by MSU was further investigated. The results showed that EC concentration-dependently improved the cell viability of LPS and MSU stimulated THP-1 cells, and significantly alleviated MSU-induced ankle edema in mice in a dose-dependent manner. In addition, EC inhibited the infiltration of inflammatory cells and local cascular congestion in ankle joint tissue. Furthermore, the secretion of inflammatory cytokines (IL-1β, IL-18, IL-6, and TNF-α) activation of NLRP3 inflammasome and NF-κB signaling pathway were markedly suppressed by EC *in vitro* and *in vivo*.

**Conclusion:** These results indicated that EC could effectively improve MSU-induced acute gouty arthritis via inhibiting NLRP3 inflammasome and the NF-κB signaling pathway *in vitro* and *in vivo*, which suggested that EC might be a promising active ingredient for the prevention and treatment of gouty arthritis.

## Graphical Abstract

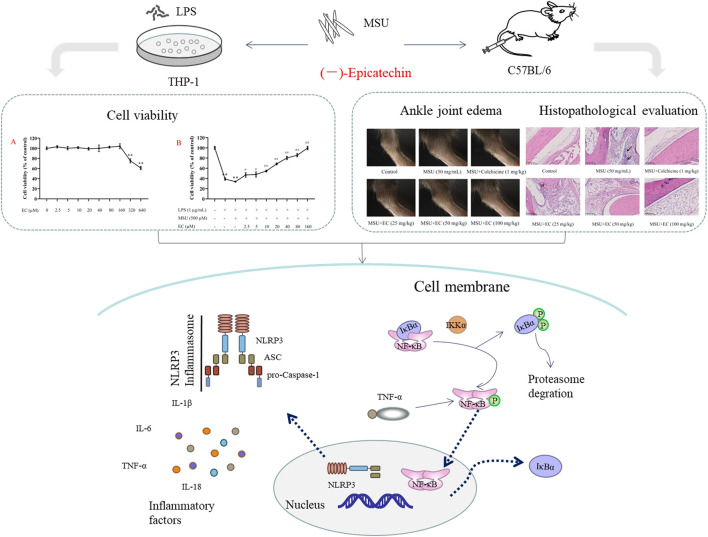

## Introduction

Gouty arthritis is an inflammatory disease caused by the disorder of purine metabolism and reduction of uric acid excretion, which can lead to the deposition of monosodium urate (MSU) crystals in joints and surrounding tissues (Cleophas et al., 2017; Ragab et al., 2017). It is common in human limbs, accompanied by main clinical manifestations including rubor, swelling, heat and pain, etc. Once the cells or tissue damaged, uric acid or MSU immediately released. As a kind of damage associated molecular patterns (DAMPs) (Vénéreau et al., 2015), uric acid or MSU could be recognized by intracellular receptor, such as nucleotide oligomerization domain-like receptor (Rock et al., 2013). In addition, MSU could directly activate NLRP3 inflammasome (Yang et al., 2020), thereby activating Caspase-1 and cutting pro-IL-1β and pro-IL-18, resulting in the release of IL-1β, IL-18 and IL-6, ultimately triggering inflammatory response (Yang et al., 2020; Leng et al., 2019). Moreover, MSU could also be recognized by Toll-like receptors, leading to the activation of NF-κB signaling pathway (He et al., 2019). Then inflammatory mediators and adhesion molecules related to the inflammatory immune response were initiated, and transcription of inflammatory factors (IL-1β, IL-6, and TNF-α) were up-regulated, resulting in continuously activation of NF-κB cascade and aggravation inflammatory response (He et al., 2019; Mylona et al., 2012).

In recent decades, incidence rate of gout has been substantially rising which was proved to associate with people’s diet and lifestyle, such as high protein, high purine compounds and high-stress lifestyle (Wilson and Saseen, 2016; Terkeltaub, 2017). Conventional drugs (non-steroidal anti-inflammatory drugs, colchicine, etc.) could quickly inhibit inflammation and relieve pain in the treatment of gouty arthritis. However, adverse effects of these drugs, such as gastrointestinal reactions, leukopenia, aplastic anemia, liver damage and hair loss, have always been clinical problems, which make these drugs unsuitable for long-term use (Schlesinger, 2017). Therefore, to developing the alternatives with high efficiency and safety but low side effects are urgently needed.

Flavonoids are main active components in some medicinal plants and foods, which have a variety of pharmacological functions (Nijveldt et al., 2001). They might be beneficial to gout by reducing the activity of xanthine oxidase, thus preventing the production of uric acid and mitigating inflammation in gout attack (Kelley et al., 2006; Kawakami et al., 2021). Among flavonoids, (‐)-epicatechin (EC) is considered to be an important candidate for the beneficial effects of these flavonoid rich foods (i.g., tea, cocoa, fruits, vegetables, etc) (Qu et al., 2021). It was found that EC could improve the symptoms of cardiovascular and cerebrovascular diseases, and prevent various chronic diseases (diabetes, gout, COPD, etc.), which were attributed to its outstanding antioxidant and anti-inflammatory activities (Dower et al., 2016; Qu et al., 2021; Tian et al., 2021; Wang and Cao, 2014). Although catechin compounds were effective in relieving gout (Jhang et al., 2015; Jhang et al., 2016; Lee et al., 2019), the specific mechanism of EC on acute gouty arthritis has rarely been reported.

Taken together, NLRP3 inflammasome and the NF-κB signaling pathway are of great significance to the exploration of EC on acute gout. Therefore, the aim of this study is to investigate the therapeutic effect of EC on MSU-induced acute gouty arthritis based on NLRP3 inflammasome and the NF-κB signaling pathway *in vitro* and *in vivo*, and its mechanism was further explored.

## Materials and Methods

### Materials and Reagents

(−)-Epicatechin (EC, Lot: wkq20101607) was obtained from Sichuan Victory Biological Technology Co., Ltd, (Chengdu, China), with the purity of more than 98%. Phorbol 12-myristate 13-acetate (PMA, Lot: SLBS0478V), lipopolysaccharide (LPS, Lot: 014M4019V), and monosodium urate (MSU, Lot: BC8R7559) were purchased from Sigma-Aldrich (St. Louis, MO, United States). Colchicine (Lot: G1514018) was obtained from Shanghai Aladdin Bio-Chem Technology Co., Ltd. (Shanghai, China). Penicillin–Streptomycin solution was purchased from HyClone (Logan, Utah, United States). Fetal bovine serum (FBS) was purchased from Gibco (Scoresby, Australia). Cell Counting Kit (CCK)-8 was purchased from Biosharp Life Sciences (Beijing, China). Colchicine tablets (Lot: 17EN) were purchased from Kpc Pharmaceuticals Inc. (Kunming, China) for animal experiments. The BCA Protein Assay Kit was purchased from Beyotime Institute of Biotechnology (Shanghai, China). Phosphatase inhibitor cocktail and protease inhibitor cocktail were purchased from Beijing ComWin Biotech Co., Ltd. (Beijing, China). Human-specific or mouse-specific enzyme-linked immunosorbent assay (ELISA) kits for TNF-α, IL-1β, and IL-6 were purchased from MEIMIAN (Shanghai, China). Phospho-NF-κB (p-p65), NF-κB (p65), phospho-IκBα (p-IκBα), IκBα, phospho-IKKα (p-IKKα), IKKα, NLRP3, ASC, caspase-1, β-actin, and Histone H3 antibodies were purchased from Cell Signaling Technology (Boston, MA, United States). IL-18 antibody was purchasde from proteintech (Wuhan, China). NE-PER™ Kit was purchased from Thermo Scientific (Waltham, MA, United States). Western ECL Substrate was purchased from Bio-Rad (Hercules, United States).

### Cell Culture and Treatments

Human-derived monocytic leukemia cell line THP-1 cells were purchased from Cell Bank of Chinese Academy of Sciences (Shanghai, China). They were cultured in RPMI-1640 medium containing 10% FBS and 1% bi-antibody (100 µg/ml streptomycin and 100 U/mL penicillin) at 37°C under 5% CO_2_. THP-1 cells in the logarithmic growth phase were treated with 50 ng/ml PMA for 48 h to induce the differentiation into macrophages (Shi et al., 2013; Hsieh et al., 2019; Yin et al., 2020).

### Cell Viability Assay

THP-1 cell suspension (7 × 10^4^ cells/mL) was seeded into a 96-well plate (100 µL/well) and stimulated with 50 ng/ml PMA for 48 h. Then, the cells were treated with EC of various concentrations (2.5, 5, 10, 20, 40, 80, 160, 320, and 640 μM) for 24 h to evaluate the cytotoxicity of EC. After washing with phosphate buffered saline (PBS, pH 7.4), RPMI-1640 medium containing 10% CCK-8 solution was added into each well to incubate for another 0.5 h. The optical density (OD) of each well was measured at 450 nm with a microplate reader.

Similarly, THP-1 cells were incubated with LPS (1 μg/ml) for 24 h after PMA stimulation. The cells were subsequently washed twice with PBS followed by incubation with various concentrations (2.5, 5, 10, 20, 40, 80, and 160 μM) of EC for 0.5 h and MSU (final concentration of 500 μM) for another 23.5 h. The cell viability was determined the same way as mentioned above.

The cell viability calculation formula was as follows:

$$\mathrm{Cell}\;\mathrm{viability}\left(\%\right)=\frac{OD_{\mathrm{sample}}-OD_{\mathrm{blank}}}{OD_{\mathrm{control}}-OD_{\mathrm{blank}}}\times \mathrm{100\%}$$ (1)

### MSU-Induced Acute Gouty Arthritis Model *In Vitro*

Cells (7 × 10^5^ cells/mL) with 50 ng/ml PMA were seeded in a 6-well plate (2 ml/well) for 48 h. Then, the cells were incubated with LPS (1 μg/ml) for 24 h. After removing the medium, the cells were washed twice with PBS (pH 7.4). The cells were subsequently incubated with various concentrations of EC (20, 40, 80 μM) or colchicine (0.1 μM) for 0.5 h, and incubated with MSU (final concentration 500 μM) for another 23.5 h.

### Animal and Treatments

Male C57BL/6 mice aged 5–6 weeks (18–20 g) were purchased from Shanghai SLAC Laboratory Animals Co., Ltd. (certificate number: SCXK 2017-0005) and housed in a standard environment with controlled temperature (23 ± 1°C) and relative humidity (55 ± 5%). The mice were acclimated to the environment for one week before the experiment. All mice received humane care during the study with unlimited access to chow and water. All experimental procedures were conformed to Guidelines for the Care and Use of Laboratory Animals [National Institutes of Health (NIH), Bethesda, MD, United States] and approved by the Animal Care and Use Committee of Zhejiang Chinese Medical University (Hangzhou, China. Permission number: SYXK 2018-0012).

The gouty arthritis mice model was established according to previous references (Shi et al., 2013; Yin et al., 2020) with slight optimization. In brief, 90 male C57BL/6 mice were randomly divided into six groups (15 animals per group), namely, the control group, MSU group, MSU + colchicine group, MSU + EC (25 mg/kg/d) group, MSU + EC (50 mg/kg/d) group, and MSU + EC (100 mg/kg/d) group. The mice in colchicine group were intragastrically administered with colchicine solution (1 mg/kg/d), and EC groups were given different doses of EC (Bettaieb et al., 2016; Lee et al., 2019) consecutively for 7 days. The mice in the control group and MSU group were given normal saline at the same time. One hour after intragastric administration on the 6th day, 0.025 ml MSU suspension (50 mg/ml) was injected into the right ankle of mice at 45° along the dorsal side to induce the acute gouty arthritis model. An obvious bulge was observed on the opposite side of the injection site once injection was successful. The normal group was correspondingly injected with the same volume of normal saline. The mice were euthanized by decapitation and placed on ice after 3 h of gavage on the 7th day. The skin and muscle near the ankle joint were cut and removed along the line above the ankle joint. One part of the ankle joint tissue was used for pathological section observation, and the other part was placed in a −80°C refrigerator for later use.

### Evaluation of Ankle Joint Edema

A horizontal line was drawn at 5 mm above the ankle joint with an indelible marker before MSU injection, in order to unify the measurement standard of toe volume. The toe volume of mice was measured before and 2, 4, 6, 10, and 24 h after model establishment with a toe volume measuring device (Jin et al., 2018). Swelling index was calculated according to the following formula:

$$\mathrm{Swelling}\;\mathrm{index}\left(\%\right)=\frac{V_{\mathrm{after}\;\mathrm{injection}}-V_{\mathrm{before}\;\mathrm{injection}}}{V_{\mathrm{before}\;\mathrm{injection}}}\times \mathrm{100\%}$$ (2)

All the abovementioned measurements were performed by a specified experimenter blinded to experimental conditions.

### Histopathological Assessment

The ankle joint tissue samples were collected immediately, fixed in the 4% paraformaldehyde solution, and then decalcified with EDTA embedded with paraffin. The paraffin sections were stained with hematoxylin and eosin (HE) according to the standard protocol. Then, the histopathological evaluation was performed under the light microscope (Hamamatsu, Japan) with 20× objectives.

### Detection of Inflammatory Factors

The cell supernatant was collected and centrifuged at 4°C and 3000 rpm for 10 min. The tissue of the ankle joint was grinded with liquid nitrogen, and the ratio of ankle joint powder to normal saline was 1:10 (g/ml). After grinding with a homogenizer (Roche, Germany) and centrifuging at 4°C and 3000 rpm for 10 min, the supernatant was collected. The contents of three inflammatory factors (IL-1β, IL-6 and TNF-α) in the supernatants were determined according to the instructions of ELISA kits.

### Western Blotting Analysis

THP-1 cells and the tissue of the ankle joint were collected and lysed in a RIPA buffer containing 1% phosphatase inhibitor and protease inhibitor. The total protein concentration was measured using BCA protein assay kit after centrifugation at 4°C and 12000 rpm for 10 min. After denaturation, the sample containing approximately 30 μg proteins were electrophoresed and separated in 10% or 12% polyacrylamide gel prior to being transferred onto PVDF membranes. Subsequently, the PVDF membrane containing the target protein was blocked with 5% (w/v) non-fat milk in TBST buffer for 1 h at room temperature, and incubated with corresponding primary antibodies (NLRP3, ASC, Caspase-1, IL-18, NF-κB p65, NF-κB p-p65, IκBα, p-IκBα, IKKα, and p-IKKα) at 4°C for overnight. The membranes were washed three times (5 min each time) with TBST and then incubated with the anti-rabbit IgG for 2 h at room temperature. NF-κB p65 and NF-κB p-p65 were visualized by using the enhanced chemiluminescence system (Guangzhou Boluteng, China), while other proteins were detected by using the two-color infrared laser imaging system (Gene company, America). The optical density analysis of the protein bands was performed with ImageJ analysis program.

### Nuclear Translocation of NF-κB p-p65

To investigate the effect of EC on the activation of NF-κB p-p65, the nuclear proteins of the cells were extracted according to NE-PER Nuclear and Cytoplasmic Extraction Reagents instructions. NF-κB p-p65 expression was detected through Western blotting that were operated same as mentioned above. Histone H3 was the reference protein of the nucleus.

### Statistical Analysis

The results were analyzed by one-way analysis of variance (ANOVA) followed by multiple comparisons with the Dunnett test using the statistical software of SPSS 24.0 or two-tailed unpaired Student’s t-test using GraphPad Prism 8.0. The data were shown as mean ± SEM of three independent experiments. *p* < 0.05 was considered statistically significant.

## Results

### Effects of EC on THP-1 Cell Viability

**FIGURE 1:**
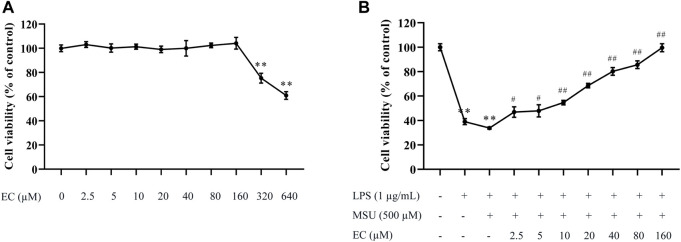
Effects of EC on THP-1 cell viability. **(A)** Effect of EC alone on THP-1 cell viability. After induction with 50 ng/ml of PMA for 48 h, THP-1 cells were treated with different concentrations of EC (2.5–640 μM) for 24 h. **(B)** Effect of EC on THP-1 cell viability stimulated by LPS and MSU. After PMA (50 ng/ml) induction for 48 h and LPS pre-stimulation for 24 h, THP-1 cells were pretreated with different concentrations of EC (2.5–160 μM) for 0.5 h followed by MSU (500 μM) stimulation for 23.5 h. Cell viability was determined using the CCK-8 assay kit. Data are represented as mean ± SEM of three independent experiments. ^**^ *p* < 0.01 compared with the control group; ^#^ *p* < 0.05 or ^##^ *p* < 0.01 compared with the LPS+MSU group.

THP-1 cells were treated with different concentrations of EC to evaluate the cell viability. As shown in Figure 1A, EC had no effect on cell viability at the concentrations of 2.5–160 μM. However, obvious cytotoxicity was observed when the concentration of EC was above 160 μM. Therefore, the EC concentration range of 2.5–160 μM was used in further experiments.

Subsequently, we investigated the protective effect of different concentrations of EC on THP-1 cells stimulated by LPS and MSU. The results are shown in Figure 1B. EC could concentration-dependently improve cell viability, especially for the concentrations between 20–160 μM whose cell viability increased by more than 50%. Thus, 20, 40, and 80 μM were chosen as the concentrations for further experiments.

### EC Inhibited MSU-Induced Overexpression of Inflammatory Cytokines in THP-1 Cells

**FIGURE 2:**
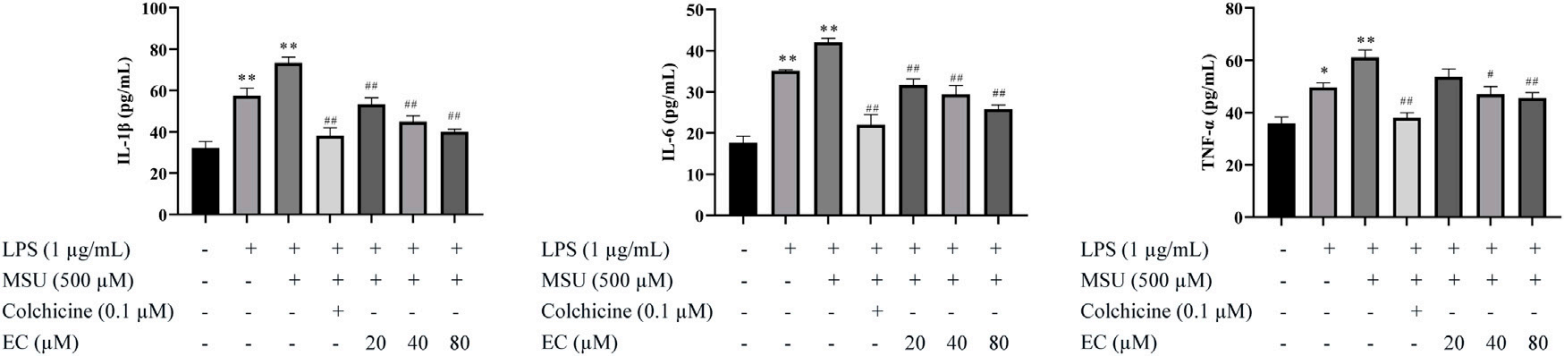
Levels of IL-1β, IL-6, and TNF-α in THP-1 cell supernatants. After PMA (50 ng/ml) induction for 48 h and LPS (1 μg/ml) pre-stimulation for 24 h, THP-1 cells were pretreated with or without colchicine or various concentrations of EC for 0.5 h followed by MSU (500 μM) stimulation for 23.5 h. Data are represented as mean ± SEM of three independent experiments. ^*^ *p* < 0.05 or ^**^ *p* < 0.01 compared with the control group; ^#^ *p* < 0.05 or ^##^ *p* < 0.01 compared with the LPS+MSU group.

**FIGURE 3:**
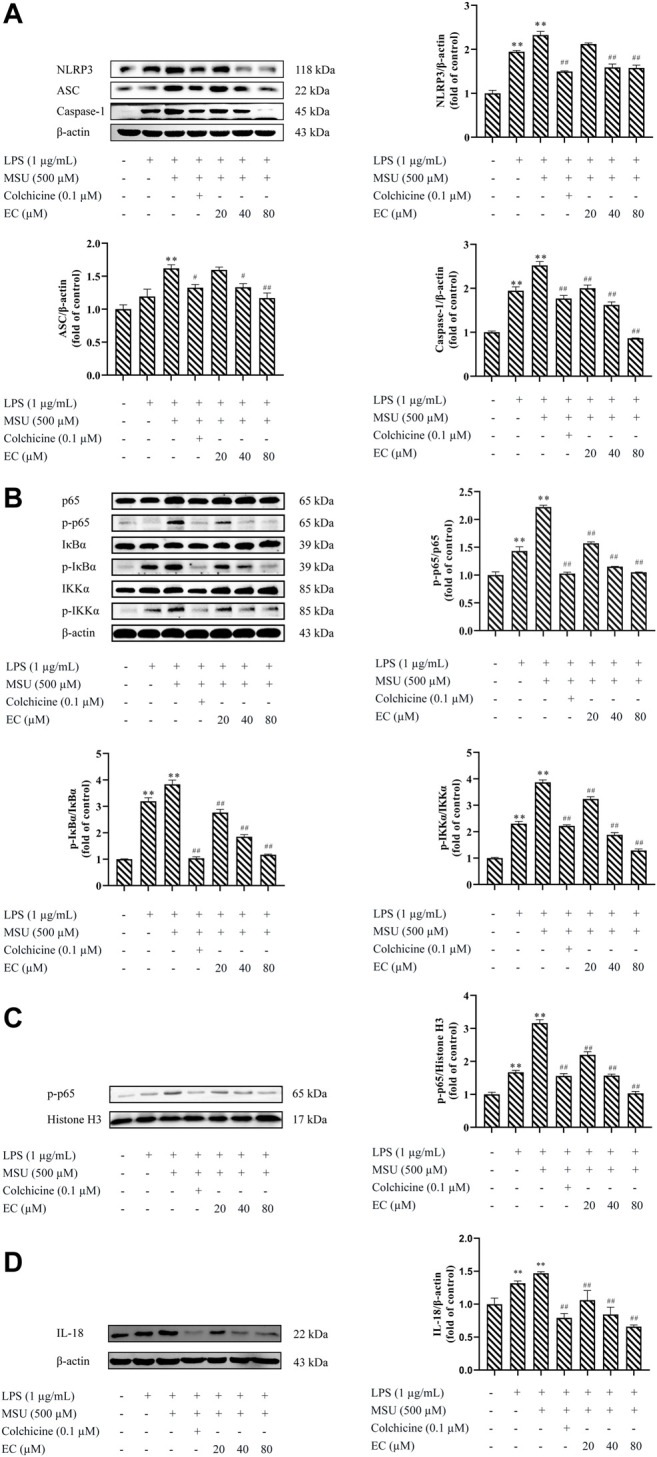
Inhibitory effect of EC on MSU-induced NLRP3 inflammasome and NF-κB pathway-related protein expression *in vitro.* **(A)** The protein expressions of NLRP3 inflammasome components in THP-1 cells. **(B)** Related proteins expressions of the NF-κB signaling pathway. **(C)** Expression of NF-κB translocation. **(D)** IL-18 expression in THP-1 cells. Data are represented as mean ± SEM of three independent experiments. ^**^ *p* < 0.01 compared with the control group; ^#^ *p* < 0.05 or ^##^ *p* < 0.01 compared with the LPS + MSU group.

To evaluate the effects of EC on inflammation, inflammatory cytokines in cell supernatant of THP-1 cells were determined. As shown in Figures 2, 3D, the secretion of IL-1β, IL-6, TNF-α and IL-18 were significantly inhibited by EC in a concentration-dependent manner when comparing with LPS+MSU group. Especially, high concentration of EC exhibited best effect, which was comparable to the effect of colchicine.

### EC Suppressed MSU-Induced Activation of NLRP3 Inflammasome and the NF-κB Pathway in THP-1 Cells

Since the secretion of IL-1β and other inflammatory factors was mediated by the activation of NLRP3 inflammasome (Liu et al., 2017), we further examined the potential effect of EC on NLRP3 inflammasome in THP-1 cells. The results showed that EC dramatically inhibited the overexpression of NLRP3, caspase-1, and ASC caused by MSU (Figure 3A), indicating that the activation of NLRP3 inflammasome might be inhibited.

It was reported that TNF-α expression depended on the activation of NF-κB (Zhang et al., 2016). Upregulation of key components of NLRP3 inflammasome was observed in MSU-stimulated macrophages in this study. Therefore, we speculated whether EC exerts a protective effect by inhibiting the activation of NF-κB. As shown in Figure 3B, EC significantly inhibited p-IKKα/IKKα, p-IκBα/IκBα and p-p65/p65 in a concentration-dependent manner when comparing with that of LPS+MSU group.

To further explore whether EC exerted its anti-inflammatory effect via nuclear translocation by promoting entering of p65 into the nucleus, we tested p-p65 expression in the nucleus. The result showed that the nuclear expression of p-p65 in the EC group was significantly downregulated compared with the LPS + MSU group (Figure 3C). All the results indicated that EC suppressed the release of inflammatory factors by inhibiting the activation of NLRP3 inflammasome and the NF-κB signaling pathway.

### EC Alleviated MSU-Induced Ankle Edema in Mice

**FIGURE 4:**
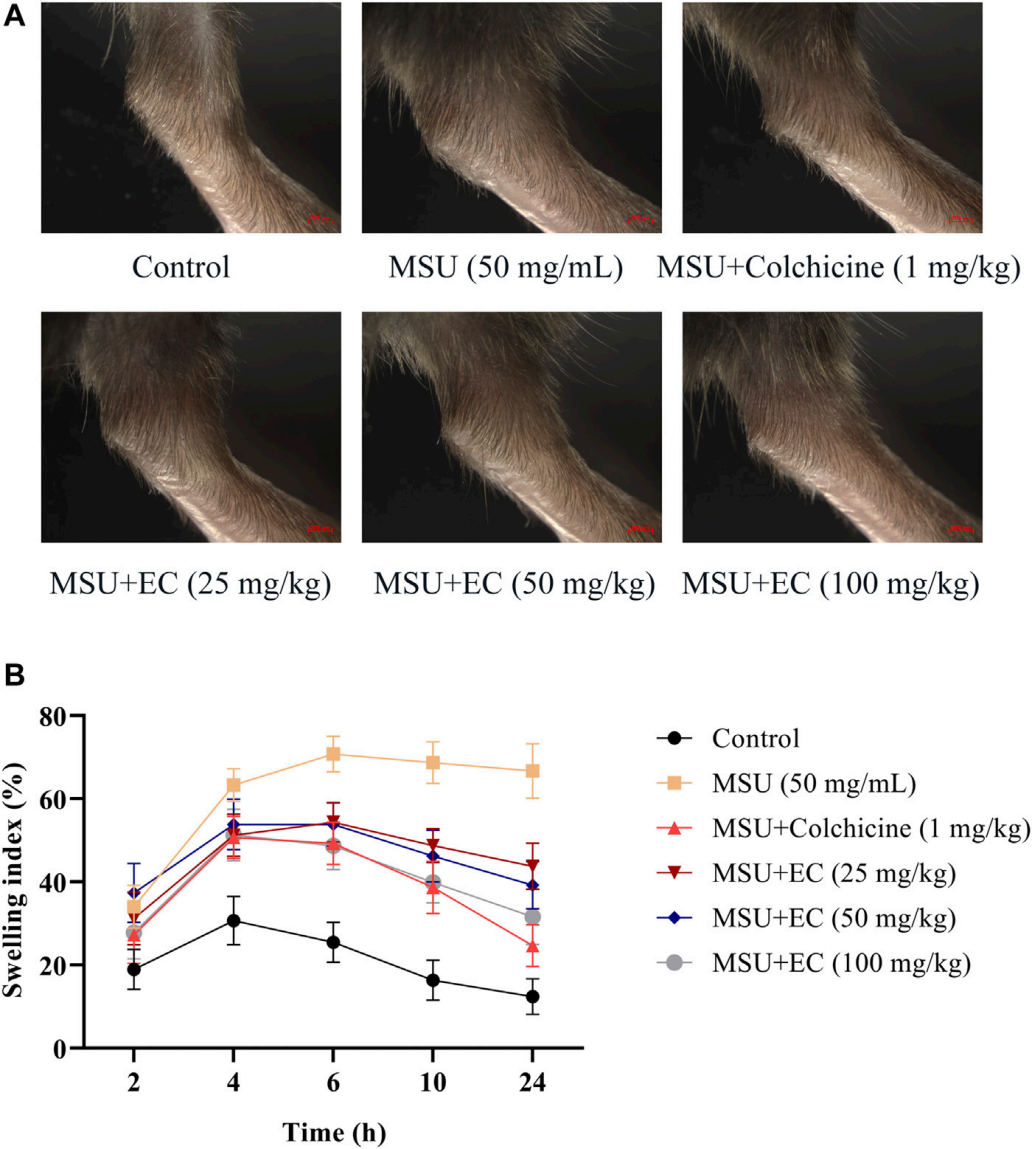
Effect of EC on ankle joint swelling in C57BL/6 mice with MSU-induced acute gouty arthritis. Mice were intragastrically administrated with different doses of EC consecutively for 7 days, followed by MSU (0.025 ml MSU suspension with 50 mg/ml) injection into the ankle 1 h after EC administration on the sixth day. The swelling degree at different times is represented as the ratio of after injection to before injection. **(A)** Representative photo of ankle joint swelling in mice 24 h after MSU injection. **(B)** The tendency of swelling degree. Data are represented as mean ± SEM of ten mice per group. ^**^ *p* < 0.01 compared with the control group; ^#^ *p* < 0.05 or ^##^ *p* < 0.01 compared with the MSU group.

The mice model of MSU-induced gouty arthritis was administrated with EC for 7°days to explore the effects of EC on gouty response *in vivo*. Obvious swelling appeared 2 h after MSU injection (Figure 4A). The swelling rate of the ankle joint in control, colchicine, and EC groups (50 mg/kg and 100 mg/kg) reached the peaks at 4 h and then decreased to some extent. However, the swelling rate in the MSU group increased continuously until 6 h after MSU injection, followed by a gradual decrease, but a far higher level was kept than other groups during the experiment (Figure 4B, Table 1). Besides, the ankle swelling rate in the MSU group was significantly increased at all experimental time nodes after MSU injection compared with the control group (Table 1) (*p* < 0.01), which indicated that the gouty arthritis model was successfully established. Interestingly, all doses of EC, especially for high dose, dramatically ameliorated MSU-induced ankle edema since 4 h.

**TABLE 1:** Measurement of the swelling degree of the C57BL/6 mice ankle joint (%) (mean ± SEM, *n* = 10).

Group	Dose	Time (h)				
		2	4	6	10	24
Control	−	18.95±6.75	30.72±8.15	25.49±6.75	16.34±6.75	12.42±6.01
MSU	50 mg/ml	34.01±7.21^**^	63.27±5.55^**^	70.75±5.96^**^	68.71±7.03^**^	66.67±9.21^**^
MSU+colchicine	1 mg/kg	27.33±9.66	50.67±7.17^##^	49.33±7.17^##^	38.67±8.78^##^	24.67±7.06^##^
	25 mg/kg	31.25±8.84	51.25±7.10^##^	54.38±6.62^##^	48.75±5.74^##^	43.75±7.80^##^
MSU+EC	50 mg/kg	37.34±9.92	53.80±7.92^##^	53.80±7.34^##^	46.20±8.67^##^	39.24±7.89^##^
	100 mg/kg	27.85±8.85	51.27±8.67^##^	48.73±8.03^##^	39.87±6.97^##^	31.65±9.34^##^

^**^ *p* < 0.01 compared with the control group.

^##^ *p* < 0.01 compared with the MSU group.

Data are represented as mean ± SEM of ten mice per group.

### EC Suppressed MSU-Induced Inflammatory Cell Infiltration in the Mice Ankle Joint

**FIGURE 5:**
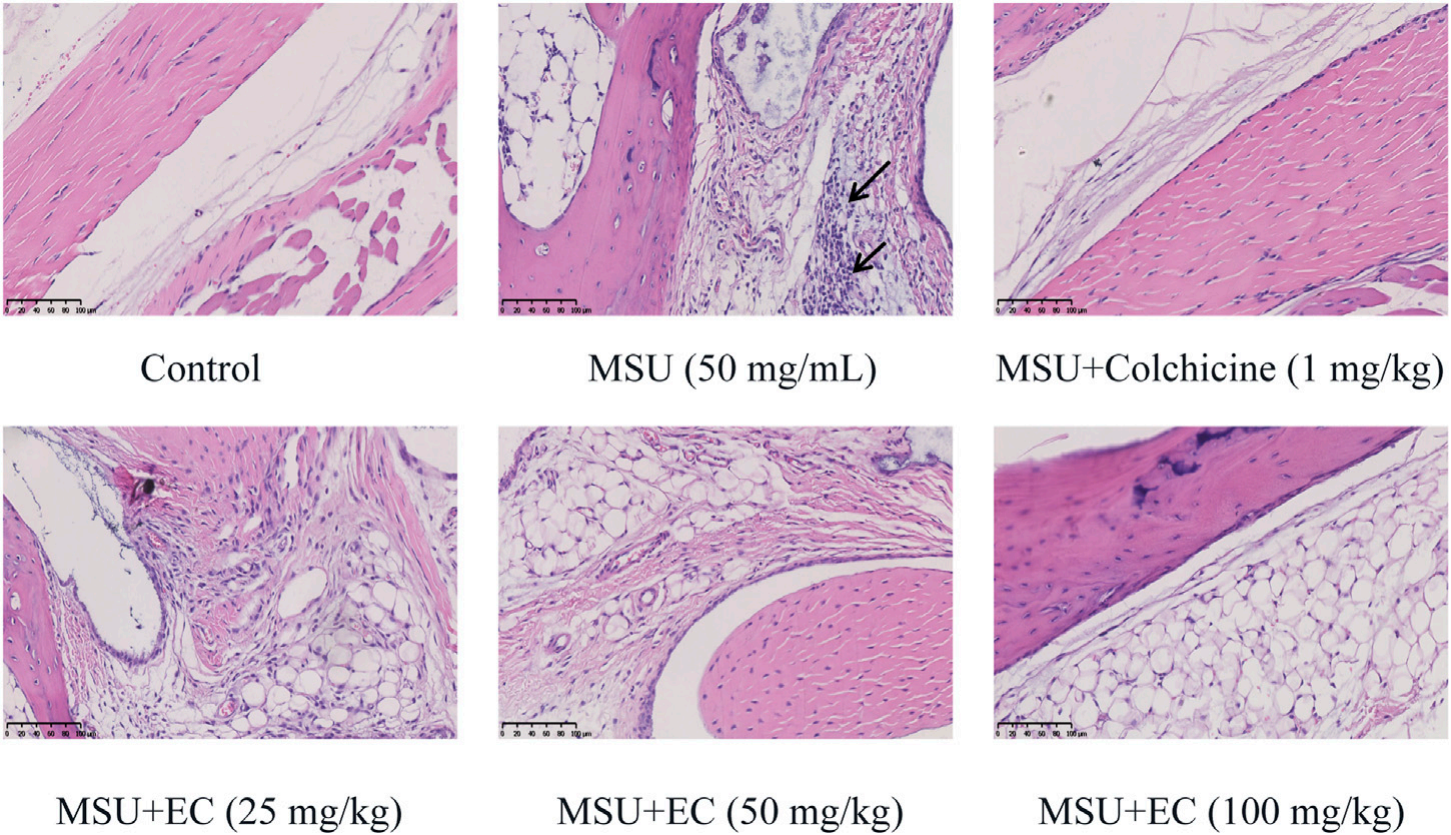
Effect of EC on the histological changes of the ankle joint 24 h after MSU injection. Mice were intragastrically administrated with different doses of EC consecutively for 7 days, followed by MSU (0.025 ml MSU suspension with 50 mg/ml) injection into the ankle 1 h after EC administration on the sixth day. The ankle joint 24 h after MSU injection was fixed in the 4% paraformaldehyde solution and stained with hematoxylin and eosin (HE). A representative HE staining image of the mice ankle joint in each group is shown (× 400, scale bar: 100 μm). The arrows represent leukocyte infiltration.

To further investigate swelling of the ankle joint in mice, histological morphology changes were observed via HE staining (Figure 5). MSU crystals significantly increased the infiltration of inflammatory cells (the blue-stained dots in the figure) into the joint tissues. The cell nucleus was significantly enlarged with the deepened staining, and the blood vessels were congested and necrotic in some areas after MSU injection. In contrast, all doses of EC administration could reduce inflammatory cell recruitment to mice ankle joints in MSU-induced gouty arthritis.

### EC Inhibited MSU-Induced Overexpression of IL-1β, IL-6, TNF and IL-18 in the Mice Ankle Joint

**FIGURE 6:**
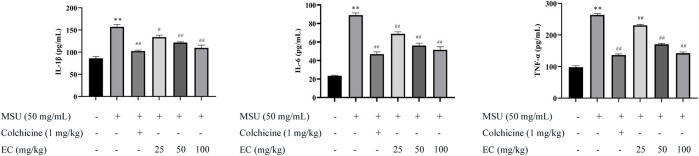
Effect of EC on IL-1β, IL-6, and TNF-α expressions in ankle joint tissues of MSU-induced acute gouty arthritis mice. Mice were intragastrically administrated with different doses of EC consecutively for 7 days, followed by MSU (0.025 ml MSU suspension with 50 mg/ml) injection into the ankle 1 h after EC administration on the sixth day. The ankle joint 24 h after MSU injection was grinded with liquid nitrogen, and the ratio of ankle joint powder to normal saline was 1:10 (g/ml). The supernatant is used for detection. Data are represented as mean ± SEM of ten mice per group. ^**^ *p* < 0.01 compared with the control group; ^#^ *p* < 0.05 or ^##^ *p* < 0.01 compared with the MSU group.

**FIGURE 7:**
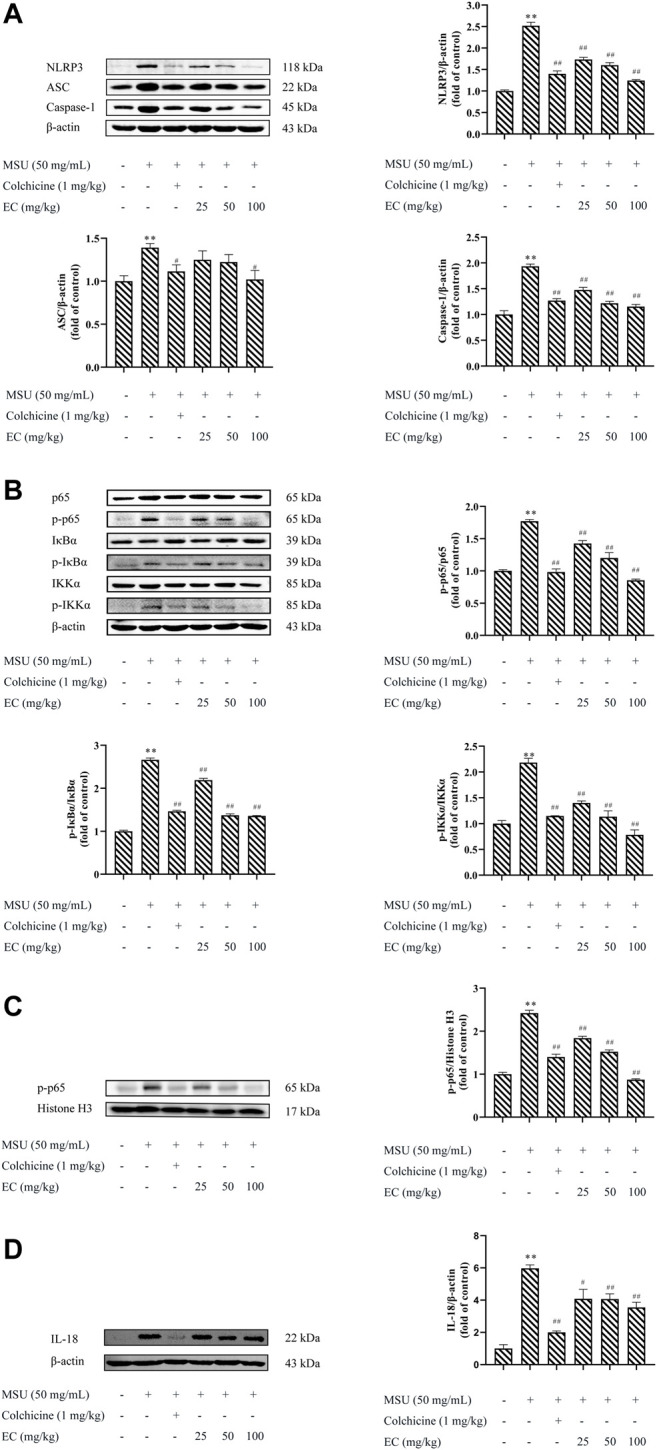
Inhibitory effects of EC on the protein expressions of NLRP3 inflammasome and the NF-κB pathway in the MSU-induced mice ankle joint. Mice were intragastrically administrated with different doses of EC consecutively for 7 days, followed by MSU (0.025 ml MSU suspension with 50 mg/ml) injection into the ankle 1 h after EC administration on the sixth day. The ankle joint 24 h after MSU injection was collected and then detected by western blotting. **(A)** The protein expressions of NLRP3 inflammasome components. **(B)** Related protein expressions of the NF-κB signaling pathway. **(C)** Expression of NF-κB translocation. **(D)** IL-18 expression in ankle joint tissues. Data are represented as mean ± SEM of three independent experiments. ^**^ *p* < 0.01 compared with the control group; ^#^ *p* < 0.05 or ^##^ *p* < 0.01 compared with the MSU group.

MSU was reported to induce the expressions of pro-inflammatory cytokines (IL-1β, IL-6, TNF-α and IL-18), resulting in the aggravation of inflammatory reaction (Yin et al., 2020; Caution et al., 2019). Similarly, the result in our study showed that MSU injection significantly increased the levels of IL-1β, IL-6, TNF-α and IL-18 in the around tissues of ankle joint (Figures 6, 7D). Interestingly, EC administration significantly reduced the expressions of these inflammatory cytokines, especially for high dose of EC, which was consistent with that in THP-1 cells. The effect of the high-dose group was comparable to that of colchicine.

### EC Inhibited MSU-Induced Activation of NLRP3 Inflammasome and the NF-κB Pathway in the Mice Ankle Joint

The effects of EC on the activation of NLRP3 inflammasome and NF-κB pathway in ankle joint were shown in Figures 7A-C. MSU significantly increased NLRP3, Caspase-1, ASC, p-p65/p65, p-IκBα/IκBα and p-IKKα/IKKα protein expressions in ankle joint tissues, which were significantly attenuated by colchicine. Similar to anti-gout agent colchicine, EC could also significantly suppress protein the overexpression of NLRP3 inflammasome and the NF-κB pathway in ankle joint tissues (Figure 7A, 7B), as well as the protein expression of p-p65 in the nucleus (Figure 7C). These results were consistent with that in THP-1 cells.

## Discussion

Gout is a common inflammatory arthritis worldwide, with a higher incidence rate in male than female (Dehlin et al., 2020). In recent years, it is promising to develop new therapeutic approach with the in-depth researches on pathogenesis of gout. Lots of literatures have established reliable models in vitro and in vivo to investigate the pathogenesis and treatment of gouty arthritis (Wang et al., 2015; Coderre and Wall, 1988). THP-1 cells, a human leukemia-derived monocyte line, were one of the cells that have been widely used in vitro to study monocyte/macrophage function and the effects of anti-inflammatory drugs (Lund et al., 2016). They could be differentiated into macrophages by PMA induction. In the presence of LPS and MSU, the differentiated THP-1 cells would synthesize and release a variety of inflammatory cytokines (Ding et al., 2016; Maess et al., 2014). In our study, high levels of IL-1β, IL-6 and TNF-α were observed in LPS and MSU induced gout in THP-1 cells, which means gout model in vitro was successful (Figure 2). Besides, an animal model of acute gouty arthritis was established by injecting MSU into the ankle joint, which could avoid the influence of uric acid oxidase in mammals (including dogs, cats, and mice, but excluding humans) (Shi et al., 2013; Jin et al., 2018). Acute gout mice model was successfully established as significant swelling appeared 2 h after MSU injection in ankle joints of mice (Figure 4A), which was consistent with previous report (Li et al., 2019).

EC, a kind of catechin compounds, has been shown to play a significant anti-inflammatory role in a variety of inflammatory diseases, including colitis (Zhang et al., 2016), atherosclerosis (Morrison et al., 2014), acute liver injury (Wu et al., 2020), obesity (Bettaieb et al., 2016), COPD (Tian et al., 2021), etc. It was reported that EGCG, rather than EC, has a significant anti-inflammatory effect in primary human rheumatoid arthritis synovial fibroblasts (Fechtner et al., 2017). (‐)-epicatechin-3-O-β-D-allopyranoside (ECAP), a glycoside, could effectively inhibit inflammatory pain and adjuvant-induced arthritis, which might be related to the inhibition of NF-κB pathway activation (Hsiao et al., 2019). In present study, we found that EC could significantly improve the cell viability of THP-1 cells induced by LPS and MSU (Figure 1), and dose-dependently reduce ankle joint swelling and leukocyte infiltration in MSU-induced gouty arthritis mice (Figures 4, 5), which was similar to that of colchicine. These data indicated that EC might possess protective effect on gouty arthritis model both *in vitro* and *in vivo*.

Immune cells such as macrophages were considered to play vital role in gouty arthritis (Li et al., 2019). LPS and MSU, an exogenous or endogenous stimulus, were recognized by macrophages and stimulated them to release IL-1β, IL-6, TNF-α and IL-18 (Lee et al., 2015; Ruiz-Miyazawa et al., 2017). IL-1β is a key cytokine that could initiate neutrophil recruitment, which lead to local or systemic inflammatory responses (Ruiz-Miyazawa et al., 2018). IL-6 was a pro-inflammatory cytokine that could promote bone damage (Cavalcanti et al., 2016). Jhang (Jhang et al., 2016) demonstrated that EGCG reduced IL-1 and IL-6 levels and ameliorated MSU-induced inflammation. While high level of TNF-α that secreted by macrophage could induce or aggravate inflammatory responses by activating neutrophils and lymphocytes, and inducing lysosome release. IL-18 was produced by the inflammasome through Caspase-1 activation. It plays an important role in the immunomodulatory process (Cavalcanti et al., 2016). In our study, we found that EC dramatically inhibited MSU-induced overexpression of IL-1β, IL-6, TNF-α and IL-18 both in vitro and in vivo (Figures 2, 3D, 6, 7D), which were consistent with the anti-inflammatory effects of EC (Ruijters et al., 2014; Wang and Cao, 2014). The results suggested that therapeutic effects of EC on gouty arthritis might be contribute to the inhibition of inflammatory cytokines, which may be related to the function of inflammasome.

To further explore the mechanism of EC treatment on gouty arthritis, the changes of NLRP3 inflammasome were investigated. NLRP3 inflammasome was considered as the main pathogenic factor in the pathogenesis of gout (Ruiz-Miyazawa et al., 2018). As DAMPs, MSU was recognized by cells and swallowed into the cytoplasm, triggering the activation of NLRP3 inflammasome (Staurengo-Ferrari et al., 2018). Activated the NLRP3 inflammasome cleaved pro-IL-1β and pro-IL-18 into mature IL-1β and IL-18 which released outside the cell, eventually resulting in aggravated inflammation and tissue damage (Huang et al., 2018; Leng et al., 2019). At the same time, Caspase-1 was cleaved and activated, leading to a conserved cell death program known as pyroptosis (Bergsbaken et al., 2009). Likewise, the results in our study showed that MSU significantly increased the protein expression of NLRP3, ASC and Caspase-1. Notably, EC could significantly reduce the expression of NLRP3 inflammasome key proteins in MSU-induced gout inflammation (Figures 3A, 7A). This was consistent with that in Figure 1, which indicated that EC improved the cell viability via preventing cell pyroptosis caused by LPS and MSU. Our results were consistent with those of Lee et al. (2019), who demonstrated that EGCG, a kind of catechin compounds, prevented acute gout by suppressing the activation of NLRP3 inflammasome in macrophages. These results suggested that EC could alleviate MSU-induced gouty arthritis via inhibiting NLRP3 inflammasome activation, thereby downregulating the expressions of inflammatory cytokines.

**FIGURE 8:**
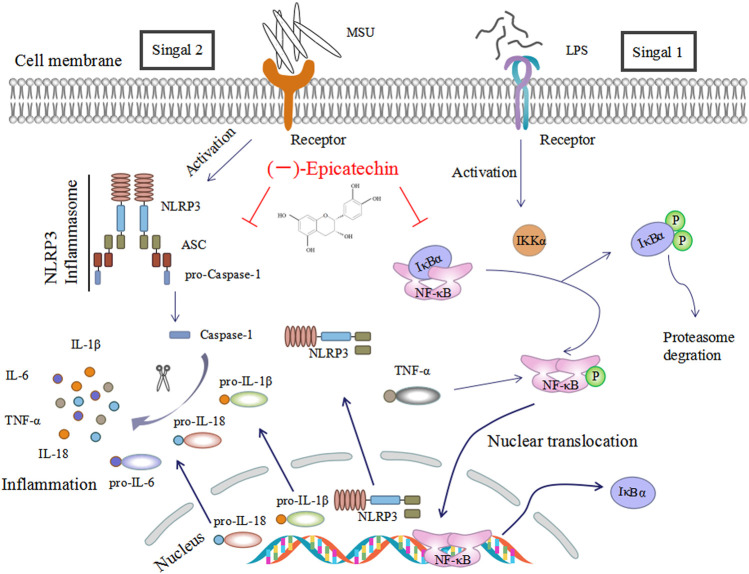
Schematic diagram of the mechanism of EC in the treatment of acute gouty arthritis. EC relieved acute gouty arthrities through inhibiting NF-κB signal transduction, weakening NLRP3 inflammasome activation and reducing IL-1β and IL-18 secretion. LPS: lipopolysaccharide; MSU: monosodium urate crystals; and EC (−)-epicatechin.

As we know, the activation of NLRP3 inflammasome involves two steps (Liu et al., 2017). The NF-κB signaling pathway, as the priming signal, is activated by different pathogen-related molecular patterns (PAMPs) (LPS, LTA, etc.), resulting in increased expression of NLRP3, pro-IL-1β and IL-18. Subsequently, NLRP3 inflammasome is activated by DAMPs (uric acid, ATP, etc.), leading to overexpression of inflammatory factors. NF-κB is the key factor in the pathogenesis of gouty arthritis. Blocking the activation of IKKs/IκB/NF-κB could inhibit the release of pro-inflammatory cytokines, including TNF-α, a downstream cytokine in the NF-κB pathway (Wu et al., 2019), and thus alleviated tissue injury (Chen et al., 2019; Ma et al., 2019; Rius-Pérez et al., 2020). Mackenzie et al (Ruiz-Miyazawa et al., 2017) demonstrated that EC inhibited PMA-induced NF-κB expression in Jurkat T cells. Similarly, our data in vivo showed that EC treatment down-regulated MSU-induced NF-κB p65 subunit translocation to the nucleus, as well as phosphorylation and degradation of IκBα (Figures 7B,C). The results added to the growing body of evidence that EC could alleviate various inflammatory diseases by inhibiting NF-κB signaling pathway (Mackenzie et al., 2004; Cremonini et al., 2018; Denise Prince et al., 2019). Taken together, our results indicated that the therapeutic effects of EC on gouty arthritis were mediated through the inactivation of NF-κB pathway (Figure 8). In addition, study (Lee et al., 2015) has shown that LPS promoted the synthesis of inflammatory factors by activating TLR4-MyD88-BLT2-Nox1-ROS-NF-κB pathway. Giving EC has good antioxidant properties, it may also exert anti-gout effects by inhibiting the production of ROS that further inhibits the cascade of NF-κB. However, relevant experiments need to be confirmed.

## Conclusion

In conclusion, our research showed that EC, as a natural polyphenol compound present in plants, could effectively prevent MSU-induced gouty arthritis *in vitro* and *in vivo*. And its mechanisms were to inhibit NF-κB signal transduction, weaken NLRP3 inflammasome activation, thereby reduce the secretion of inflammatory cytokines and inflammatory infiltration in macrophages and swelling joint synovial tissues, and eventually preventing cell pyroptosis. Hence, EC might be a new and safe candidate for the clinical prevention and treatment of gouty arthritis, as well as NLRP3-related diseases.

## Data Availability

The raw data supporting the conclusions of this article will be made available by the authors, without undue reservation.
